# Narrowing down the cause of the hard-sphere nucleation discrepancy: The free energy of precritical nuclei is consistent with predictions

**DOI:** 10.1126/sciadv.aec8906

**Published:** 2026-04-24

**Authors:** Lars Kürten, Antoine Castagnède, Frank Smallenburg, C. Patrick Royall

**Affiliations:** ^1^ Gulliver UMR 7083, CNRS, ESPCI Paris, Université PSL, 75005 Paris, France.; ^2^ Laboratoire de Physique des Solides, CNRS, Université Paris-Saclay, 91405 Orsay, France.; ^3^ Department of Physics, University of Warwick, Gibbet Hill Road, Coventry CV4 7AL, UK.

## Abstract

Predicting the rate of crystal nucleation is among the most substantial long-standing challenges in condensed matter. In the system most studied (hard-sphere colloids), the discrepancy between experiments and computer simulations is more than 10 orders of magnitude. The situation with other materials (such as water) is no better. Here, we address this challenge with two developments. First, our work is a marked improvement in the precision of mapping the state point of experiments to simulation. For this, we used a combination of novel machine-learning methods for particle tracking and higher-order correlation functions. Second, we consider the free energy of precritical nuclei using confocal microscopy. These are in agreement with computer simulation. This is the first time that such free energies have been successfully compared between experiment and simulation in any material as far as we are aware.

## Teaser

Predicting nucleation rates is a challenge, but the energy of formation of precritical nuclei in colloids can now be predicted.

## INTRODUCTION

Crystal nucleation is a truly everyday phenomenon with very wide-ranging industrial and environmental implications from the purification of chemicals to crystallization of aerosol droplets, which is a major uncertainty in climate change modeling (*1*, *2*). In the case of water and noble gases, for example, even advanced state-of-the-art measurements can be hampered by the small size of liquid droplets, the effects of pressure, and extrapolation of quantities such as the interfacial tension to far-from-equilibrium conditions (*3*–*5*). It is clearly important to be able to make meaningful predictions of the rate of nucleation of crystallites. The best-known theory, classical nucleation theory (CNT) is widely accepted to be physically reasonable at a qualitative level in many cases, and, sometimes, quantitative predictions can be made, for example, in the case of confined sodium chloride solutions (*6*). However, it has a number of shortcomings (such as assuming bulk thermodynamic properties for microscopic nuclei), which make quantitative prediction challenging (*1*), and it is often fitted to experimental data a posteriori (*4*, *5*).

Computer simulation naturally addresses many of the limitations of CNT but brings with it the requirement that the model used for the material in question must be very accurate and the rare event of nucleation is often treated with some form of enhanced sampling (*1*, *7*). For example, prediction of the nucleation rate using classical models of water shows discrepancies, which may be attributed to inaccurate thermodynamic properties (*8*) and the sampling method used (*9*, *10*). Meanwhile, ab initio methods whose accuracy can be captured with machine learning (*11*–*13*), nevertheless, predict phase boundaries with limited accuracy with clear consequences for the prediction of nucleation rates (*14*). In the case of electrolyte solutions such as sodium chloride, quantitative agreement between computer simulation and experiment is very challenging, often needing a rescaling of the state point (*6*, *15*, *16*). There is also a substantial discrepancy in nucleation kinetics between different implementations of the same model of NaCl (*17*).

In such general challenges, model experimental systems such as colloids play an important role as their simple and classical interactions make accurate large-scale computer simulations possible. Moreover, their kinetics are convenient from an experimental perspective (*18*, *19*), and particle-resolved studies can provide insight into the nucleation mechanism (*20*–*22*). The kinetics are controlled by the Brownian time taken by an isolated sphere (of diameter σ) to diffuse by its own radius $\tau_{B}=3{\text{πησ}}^{3}/8k_{B}T$ where η is the solvent viscosity. This means that different sized particles may diffuse at very different rates, yet the interactions can be unchanged.

**Fig. 1.**
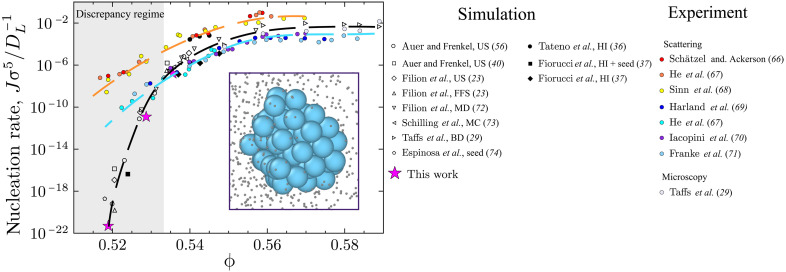
Nucleation rate discrepancy and precritical nucleus. Shown are reduced nucleation rates from experiments (*29*, *66*–*71*) and computer simulations (*23*, *29*, *36*, *37*, *40*, *56*, *72*–*74*). Experiments are divided into two branches. Top branch (orange) shows results of experiments with some sedimentation and bottom branch (blue) with almost no sedimentation. Inset shows rendering of coordinates from the experiments in this work (ϕ = 0.54) with precritical nuclei (turquoise) identified with $q_{6}$ bond-orientational order parameters of ten Wolde *et al.* (*51*). Fluid particles (gray) are depicted smaller. The methods used for simulations are: umbrella sampling (US), forward flux sampling (FFS), molecular dynamics (MD), Monte Carlo (MC), seeding (seed), Brownian dynamics (BD), and hydrodynamic interactions (HI).

However, as shown in Fig. 1, in the most studied system, colloidal hard spheres, the discrepancy between experimental measurements and prediction from computer simulation is very large and addressing it forms the topic of this work (*19*, *23*–*25*). This discrepancy is important beyond the immediate application of nucleation because the rare event sampling methods required here are used across the molecular sciences from physics to biochemistry (*26*–*28*). Direct comparison of rare event sampling is seldom possible with the level of detail available here, which renders solution of this large discrepancy all the more urgent.

In colloidal hard spheres, the nucleation rate vanishes at the freezing boundary and increases markedly upon raising the chemical potential (or volume fraction). Here, two regimes can be identified as shown in Fig. 1. At high supersaturation, where nucleation is rapid, experiment and simulation agree reasonably well. At low supersaturation where nucleation is very slow, a huge discrepancy emerges as shown by the shading in Fig. 1. The experiments in this discrepancy regime were carried out using light scattering, in which the number and size of the nuclei are inferred indirectly (*19*, *25*). More recent work using confocal microscopy where the coordinates of the particles can be tracked enables one to measure more precisely the nuclei that form (*21*) and to compare directly with predictions from simulation (*29*). Alas, while such data offer impressive insight (*30*), it comes at a high price: Colloids that are large enough to track (typically 2 to 3 μm in diameter) have a Brownian time $\tau_{B}∼10\;s$, rendering rare events like nucleation in weakly supersaturated suspensions inaccessible on reasonable experimental timescales. It is therefore hard to access the regime where the discrepancy is found using particle resolved studies with confocal microscopy.

There are two possible reasons for the discrepancy: Either the nucleation rate is different between experimental measurements and predictions based on computer simulations or the state point is not mapped correctly between the two. Until very recently (*31*), accurate determination of the state point, i.e., the volume fraction ϕ of an experimental colloidal system, has been very difficult (*32*, *33*). We note that a mechanism has been put forward, which suggests that the light scattering measurements may overstate the number of nuclei (*34*), although whether this could explain the vast discrepancy remains unclear (*19*). Other possibilities that have been investigated include electrostatic charge (*35*) and the role of hydrodynamic interactions between the colloids due to the liquid solvent (*36*–*38*), which may influence the higher-order structure of the metastable colloidal fluid (*37*, *39*), but these have been found to have too small an effect to resolve the discrepancy. The effect of polydispersity in the sizes of the colloidal particles was also investigated but this did not resolve the discrepancy (*40*). One intriguing possibility is sedimentation due to the density mismatch between the colloids and the solvent. This can lead to a density gradient and significantly enhances the nucleation rate (*41*, *42*). However, under the conditions of the previous measurements (large systems, weak sedimentation) where batch sedimentation occurs (*43*), we do not expect the colloid volume fraction to change in the central region of the sample where the measurements were made.

Here, we address the nucleation rate discrepancy with two developments using real-space studies of colloids with confocal microscopy. We carefully match the density of solvent and colloids to remove any effects of sedimentation. First, we consider the nucleation barrier. In CNT, the nucleation rate

$$J=f^{+}\rho_{f}{\mathrm{Ze}}^{-\Delta G^{*}/k_{B}T}$$ (1)

where the attempt frequency is the product of the single-particle attachment rate at the top of the barrier $f^{+}$ and the number density of the fluid $\rho_{f}$. The Zeldovich factor *Z* accounts for the probability that a critical nucleus will grow to become a macroscopic nucleus, and $\Delta G^{*}$ is the free-energy barrier of the critical nucleus (*44*). The attempt frequency $f^{+}$ is reasonably well matched between experiment and prediction. In any case, it does not vary enough numerically to account for the magnitude of the discrepancy (*45*). We therefore focus on the barrier height $\Delta G^{*}$. Although confocal microscopy can identify critical nuclei in the regime where nucleation is rapid (*21*, *29*, *42*), here, our focus is on the discrepancy regime where critical nuclei are large and exceedingly rare. Therefore, we seek to measure the free energy of formation of smaller, precritical nuclei, whose occurrence is sufficiently frequent to be accessible in experiments using confocal microscopy. While matching the free energies of such precritical nuclei between experiment and simulation does not by itself resolve the nucleation discrepancy, it does narrow down its causes, a point to which we return in the discussion.

Second, we aim to map state points between experiment and simulation with hitherto unprecedented precision. To do so, we improve the particle tracking with recently developed methods that implement machine learning (*46*) and use higher-order correlation functions (*47*) that are more sensitive to volume fraction than pair correlation functions such as the radial distribution function *g*(*r*). We calculate the effective free energy of the (precritical) nuclei as a function of size from their concentration in supersaturated fluids. We find very good agreement with computer simulations when taking into account the remaining experimental errors. This is the first time that the free energy of formation of nuclei has been successfully compared between experiment and simulation in any material as far as we are aware. Within the accuracy of our experiments, we find no discrepancy with respect to our hard-sphere simulations.

## RESULTS

Here, we briefly outline our methodology, referring the reader to Materials and Methods for further details. We used sterically stabilized polymethyl methacrylate (PMMA) spheres with a diameter of 2.0 μm and polydispersity of 4% (*48*). The particles are dispersed in a refractive index and density matching solvent mixture with salt added to screen electrostatic interactions. To extract particle coordinates from the confocal images, we used a deep learning approach called “Colloidoscope” (*46*). This particle-tracking routine was explicitly developed to track colloidal particles in crowded environments where conventional approaches based on the Crocker-Grier algorithm (*49*), for example, reach their limits. Colloidoscope was tested on the same system that we use here (*46*) and allowed us to access the coordinates of around 98% of the particles in the sample with a tracking error of 5% of the particle diameter.

To compare our experimental results to ideal hard spheres, we perform both unbiased and biased simulations of polydisperse hard spheres. We match the experimental system with a Gaussian distribution with 4% polydispersity. For the unbiased simulations, we use event-driven molecular dynamics simulations (*50*) of $N=10^{5}$ particles, in which we monitor the distribution of sizes of crystalline nuclei using the $q_{6}$ bond-orientational order parameters (*51*).

We also perform biased Monte Carlo simulations in the isobaric-isothermal ensemble (*52*), using the number of particles *n* in the largest crystalline nucleus as our bias parameter and relying on the same method for nucleus identification as in the unbiased simulations. The umbrella sampling simulations allow us to reconstruct the free-energy barriers as a function of nucleus size at different supersaturations from which we can obtain the nucleation rate.

As an additional point of comparison, we also performed simulations of particles interacting via a hard-core Yukawa interaction to test for the effects of residual electrostatic interactions between the particles. Here, we set the interaction parameters (colloid charge *Z* = 500, inverse Debye length κσ = 20) to values within the range expected for this system (*53*).

To map the state point between the experiment and simulation, we proceed as follows. As the colloids have some degree of softness (*33*), we seek the effective hard-sphere volume fraction $\phi_{\text{eff}}$. We compare the total pair correlation function h(r)=g(r)−1 as shown in fig. S1. Because *h*(*r*) depends on the volume fraction, we obtain a first estimate of the true effective volume fraction $\phi_{\text{eff}}^{\text{true}}$. When we count the particles, because some are not tracked, we obtain a smaller volume fraction $\phi_{\text{eff}}^{\text{count}}\leq \phi_{\text{eff}}^{\text{true}}$. Henceforth, we consider the effective volume fraction only and we drop the subscript.

**Fig. 2.**
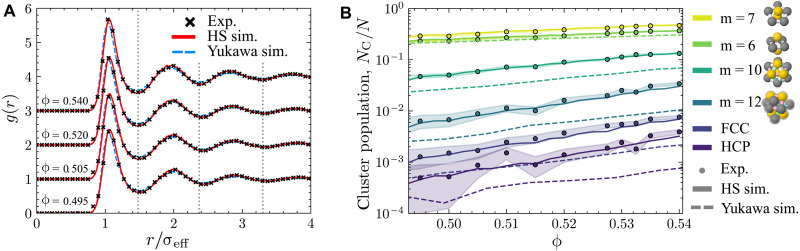
Mapping between experiment and computer simulation at fluid state points around the coexistence regime. (**A**) Radial distribution functions at a range of volume fractions. Data are offset for clarity. (**B**) Higher-order correlations in the hard-sphere fluid as a precise probe of state point matching. Selected clusters are plotted as a function of volume fraction. Errors were calculated from the SD of mean populations of different experiments mapped to the same volume fraction. HS sim., hard-sphere simulation. FCC, face-centered cubic; HCP, hexagonal close-packed.

Our second comparison is to use the population of certain clusters with a low free energy (*54*) identified with the topological cluster classification (TCC) (*47*) as shown in Fig. 2B. We weight the TCC populations and the *h*(*r*) comparisons to arrive at an optimal mapping. Because, in the experiments, not all particles are tracked and there is some uncertainty in their positions, it is important to take these errors into account when comparing against the simulations. We therefore add uncertainty in the position and remove some particles from the simulation data when we compare with the experiments. Further details are provided in Materials and Methods.

### Mapping experiments to hard-sphere state points

We compare the experimental and simulated state points using the radial distribution function *g*(*r*) in Fig. 2A. Here, we plot simulations using both hard spheres and the hard-core Yukawa model in addition to the experiments. The agreement is excellent, especially with hard spheres, indicating that the state point is well matched. This is replicated in the total correlation function shown in the Supplementary Materials.

However, the *g*(*r*) is not a strong function of volume fraction in this regime. It is desirable to have a more precise probe of volume fraction, and, here, we turn to higher-order correlations. It is possible to determine populations of clusters that minimize the local free energy of hard spheres (*54*), and the populations of some of these are shown in Fig. 2B. Overall, the agreement between experiment and hard-sphere simulation is very good, providing further evidence that the state point has been well matched. The hard-core Yukawa simulations have a noticeably lower population of the larger cluster sizes (m≥10) and, hence, are not compatible with our experimental observations. This illustrates the added benefit of the TCC comparison to the *g*(*r*) results, which are very similar between the different state points considered. Henceforth, we do not consider the hard-core Yukawa model and focus on the hard spheres only.

### Free energy of precritical nuclei

Having mapped the state point between experiment and simulation, we now move on to consider the main result, the free energy of formation of precritical nuclei. This is given by

$$\Delta G\left(n\right)/k_{B}T=-\text{log}\left(N_{n}/N\right)$$ (2)

where $N_{n}$ is the number of nuclei of size *n* and *N* is the total number of particles sampled.

We proceed as follows to determine the free energy of formation. To find the number of particles in a crystalline environment in a given nucleus, we seek an order parameter that allows us to distinguish these crystal particles from those in a fluid environment. To do so, we use the bond-orientational order parameter $q_{6}$ as described in Materials and Methods (*55*). Nuclei are then connected regions of particles in crystalline environments.

However, as mentioned above, our experiments do not track 100% of the particles, and there is some uncertainty in their positions. Because we reproduce these errors in our simulations, the observed number of particles in crystalline environments $n_{\text{obs}}$ is different to the error-free true case. This leads to the observed free energy of formation of nuclei $\Delta G_{\text{obs}}\left(n_{\text{obs}}\right)$.

**Fig. 3.**
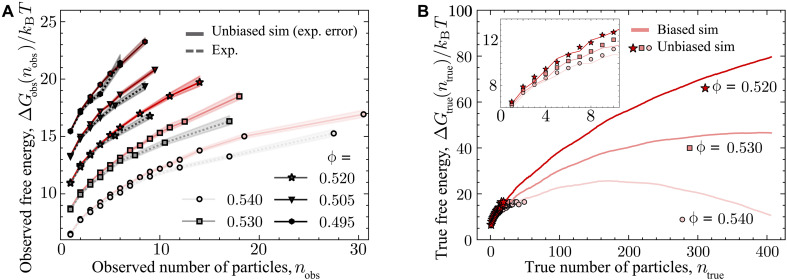
Free energy of precritical nuclei. (**A**) Comparison of the start of the observed nucleation barrier calculated from size distribution of precritical nuclei from experiments and unbiased hard-sphere computer simulations with experimental error ($\Delta G_{\text{obs}}$). Data points for both were binned into different nuclei sizes to compensate for fluctuations in the number of identified nuclei. The error bars are calculated as the square root of the number of nuclei in each bin. Data are offset by $2k_{B}T$ each for clarity. (**B**) Comparison of nucleation barriers from biased hard-sphere computer simulations (solid line) and nucleation barriers calculated from precritical nuclei in unbiased hard-sphere computer simulations (data points), both without experimental error ($\Delta G_{\text{true}}$). Inset shows zoomed-in start of the barriers.

In Fig. 3, we show the precritical behavior of the observed nucleation barrier for five representative state points in the coexistence regime. Additional state points are shown in the Supplementary Materials. For the lowest volume fractions, the agreement between experiment and simulation is very good. For ϕ=0.495,0.505,and 0.520, we can identify no meaningful disagreement between experiment and simulation. As shown in Fig. 1, this largely accounts for the regime of the nucleation rate discrepancy.

For the highest two volume fractions ϕ = 0.530 and 0.540, there is a small difference between experiment and simulation of around $k_{B}T$. We emphasize that this is very much less than that implied in Fig. 1. All other contributions being equal, the discrepancy in nucleation rates implied by a difference of $k_{B}T$ is merely a factor of e≃2.718, which is so small as to be irrelevant in the context of the hard-sphere nucleation discrepancy (Fig. 1). These small differences may reflect minor uncertainties due to mapping our experimental data to simulation. In any case, this emerges at higher volume fraction ϕ≥0.530 rather than in the regime of the discrepancy ϕ≲0.530.

### Using our results to predict nucleation rates

We can use the barriers that we determine from our umbrella sampling in Fig. 3B to estimate nucleation rates for the polydisperse systems, analogous to the work of Auer and Frenkel (*40*). To this end, we write the Zeldovich factor (*23*, *56*)

$$Z=\sqrt{\frac{|\Delta G^{\prime \prime }\left(n^{*}\right)|}{2\pi k_{B}T}}$$ (3)

Here, $G^{\prime \prime }\left(n^{*}\right)$ is the second derivative of the nucleation barrier, evaluated at the critical cluster size $n^{*}$. Following (*56*), we obtain the attachment rate $f^{+}$ from short Monte Carlo simulations at the top of the nucleation barrier by measuring the mean squared displacement of the cluster size. The resulting rates are plotted in Fig. 1 as magenta stars. This underlines the result that the precritical nuclei in our experiments are in agreement with computer simulation.

### Properties of precritical nuclei

**Fig. 4.**
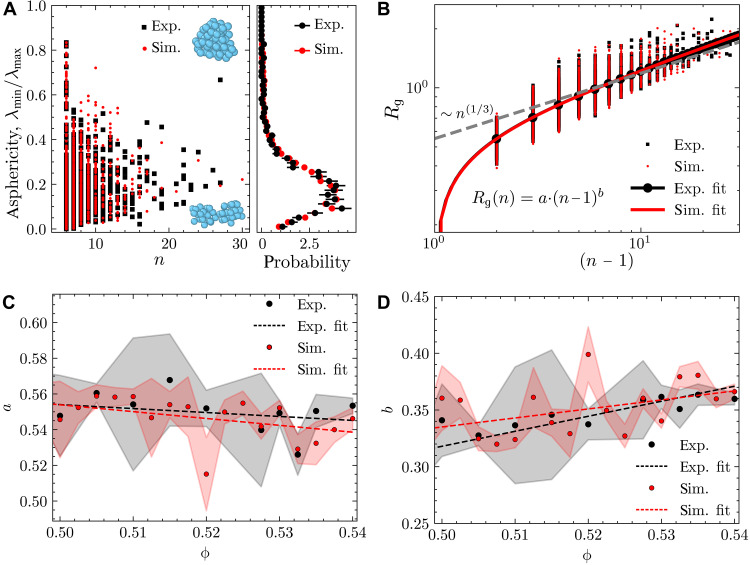
Asphericity and radius of gyration. (**A**) Asphericity quantified by the ratio of minimum and maximum variance along different axes of the precritical nuclei for hard-sphere simulations and experiments. Renderings show typical examples for quite spherical (top) and highly aspherical nuclei (bottom). (**B**) Radius of gyration as a function of the nucleus size. Fit of shape $R_{g}\left(n-1\right)=a{\left(n-1\right)}^{b}$ for experiments: a=0.552±0.005,b=0.361±0.006 and computer simulations: a=0.550±0.005,b=0.363±0.006. (**C** and **D**) Compactness as a function of volume fraction. Parameters *a* and *b* of the power law fit $R_{g}\left(n\right)=a\cdot {\left(n-1\right)}^{b}$ for experiments and simulations as a function of the state point.

Our study naturally provides a set of crystal nuclei particle coordinates, along with those of the surrounding fluid. We now proceed to analyze the properties of these nuclei. In Fig. 4A, we show the asphericity of the nuclei. The calculation is based on a simple principal components analysis (PCA) and is discussed in Materials and Methods. We see a wide distribution in the shape of the precritical nuclei, as shown by the renderings in Fig. 4. In terms of the asphericity, experiment and simulation are very well matched. Larger nuclei tend to be more spherical.

Another important geometric quantity is the compactness of the nuclei. This has been related to the crystal-fluid interfacial free energy (*57*). Like previous work (*57*), we see a considerable spread in the values of the radius of gyration for a given nucleus size as shown in Fig. 4B. We emphasize the agreement between experiment and simulation here. We also analyzed the influence of a change in volume fraction on the averaged radius of gyration. Figure 4 (C and D) shows the parameters of the fitted power law as a function of the state point.

While the data show considerable fluctuations and more statistics would be needed before strong conclusions can be drawn, there is some evidence that, as we move to higher volume fractions, we see an overall decrease in the radii of the nuclei of a given number of particles (indicated by a decrease in prefactor *a*, see Fig. 4A). This can be readily understood from the higher pressure of the surrounding fluid, which will lead to more compressed clusters. At the same time, we see a weak increase of the exponent *b* (see Fig. 4A), corresponding to a decrease in the fractal dimension 1/b of the cluster. While this effect is weak and would need stronger statistics to draw firm conclusions, this observation is consistent with past observations that nuclei of hard spheres become more fractal-like at high packing fractions (*58*). However, we emphasize that this observation should be treated as preliminary.

## DISCUSSION

We have measured the fluid structure and the free energy of precritical nuclei in colloidal hard spheres. Within our measurement error, we see no significant difference between experiment and simulation. Of course, our data do not measure the height of the critical nucleus barrier $\Delta G^{*}$, and, thus, some caution should be exercised in our results. It is possible that the small discrepancy that we find between experiment and simulation could increase for larger nuclei. However, it is important to note that the precritical barriers are controlled by the same physics that shapes the rest of the barrier. Specifically, the free-energy cost of creating a cluster of any size necessarily results from the free-energy drop associated with forming a small amount of crystal and the cost of creating the interface. The former is purely a function of the supersaturation of the fluid, while the latter results from the interfacial free energy of the fluid-crystal interface and likely only weakly depends on the interfacial curvature and roughness. The good agreement between our experiments and simulations suggests that, at least for the cluster sizes that we can access here, the hard-sphere model accurately describes the free-energy balance found in the experimental system. To put it another way, the difference in barrier height would need to be $\delta G\approx 23k_{B}T$ in the case of a discrepancy of $J_{\text{exp}}/J_{\text{sim}}=10^{10}$, which seems to imply a very strong divergence of the data in Fig. 3A. Therefore, under the assumption that the findings continue to hold for critical nuclei, it is tempting to speculate as to why in our results the discrepancy has all but disappeared. It is also quite possible that, even for postcritical nuclei, no discrepancy would be found.

There are two key differences between our work and the earlier experiments. First, we have used real-space confocal microscopy to measure the nuclei. Our data further benefits from the latest improvements in particle tracking made possible by machine learning (*46*). The coordinate nature of our data means that the analysis is essentially identical between experiment and simulation, with few assumptions being made. Second, we have matched the state point to the simulations carefully, by exploiting our real-space data with higher-order correlation functions. We have also carefully matched the density of the colloids and solvent, so that the effects of sedimentation may be neglected here.

Although the methodology to detect nuclei is, of course, very different with respect to the earlier experiments, we believe that the methods used there to detect nuclei are broadly sound [see (*59*) for a description of the scattering methods to determine nuclei size]. This brings us to consider the second factor, mapping the state point between experiment and simulation. While hard spheres are, of course, a model system and are well controlled, accurate determination of the (effective) volume fraction is fraught with difficulty (*32*, *33*). We believe that the method we have implemented here marks an improvement over previous determinations of the volume fraction in nucleation studies that used a determination of the phase diagram to infer the volume fraction. The method developed by Paulin and Ackerson (*60*), which underpinned the determination of ϕ in the earlier studies (*19*), is undoubtedly ingenious, but it does suffer from competing timescales of nucleation, phase separation, and sedimentation, which may couple to influence the value of ϕ obtained. One notes that the previous experimental data in the discrepancy regime are clustered around two lines (Fig. 1) and that these correspond to experimental systems with rather different sedimentation rates, hinting that such discrepancies within the experimental data might be related to these competing timescales (*19*, *39*).

While we emphasize that the agreement that we obtain in mapping of volume fraction between experiment and simulation is very good, some discussion of the error is clearly in order. We weight the mapping between that of the total correlation function *h*(*r*) (fig. S1) and the higher-order correlations expressed through the TCC cluster population. Now, if we take the absolute best agreement for *h*(*r*) (expressed through the least squares difference between the experiment and simulation plots), then we end up with a value δϕ = 0.0025 less than that we use. This lower volume fraction has a somewhat worse agreement with the TCC clusters. However, when we plot *h*(*r*) for the slightly higher volume fraction, there is no discernible change. We therefore take our error in volume fraction to be <0.0025, which is an order of magnitude better than that estimated previously (*32*) and close to the limit that can reasonably be expected with experiments on model colloids (*19*).

We now summarize the impact of our results on the hard-sphere nucleation discrepancy. The free energy of precritical nuclei is well described by computer simulation. We suggest three possibilities: Either (i) the state point was less accurately mapped in the previous experiments, (ii) there remains some difference in the free-energy barrier between the precritical nuclei that we have measured and that of the critical nuclei, or (iii) there may be a discrepancy in the kinetic prefactor $f^{+}$ in Eq. 1.

Regarding the state point (i), as noted above, previous experiments used the Paulin-Ackerson method (*60*) to determine the state point. We cannot rule out the possibility that this method introduced a systematic bias into the determination of the volume fraction. An error in absolute volume fraction of just δϕ=0.005 would be sufficient to shift the experimental data to match the simulations. This is double the maximum error that we estimate here, but small by historical standards (*32*, *33*). Similarly regarding point (ii), it is possible that the matching that we see for precritical nuclei becomes very much more pronounced for critical nuclei. Last, (iii), it seems unlikely that the kinetic prefactor can explain the discrepancy. Dynamics of hard spheres between experiment and simulation agree well across this regime as measured in numerous studies (*19*). Measurements of the attachment rate vary by at most one order of magnitude (*45*).

In conclusion, we find no meaningful discrepancy in the free energy of formation of precritical nuclei of colloidal hard spheres between experiment and computer simulation. We emphasize that we have based our results on a new and careful matching of state point using a new machine-learning method for particle tracking (*46*) combined with two-point and higher-order correlation functions (*47*, *54*). The real-space approach enables us to use the same methods of analysis for both experiment and simulation. Our results cast the spotlight on what might cause the discrepancy between the experimental measurements of the nucleation rate and the predictions from computer simulations. We have determined the state point in a new and accurate manner, which is one possibility. In addition, we cannot, at this stage, exclude the possibility that, while the free energy is matched between experiment and simulation for the precritical nuclei observed here, this ceases to be the case for critical nuclei. In the future, it would be attractive to extend the range of free energy of formation of nuclei that can be measured. This could be achieved with smaller particles amenable to nanoparticle resolved studies (*61*) or scattering. The latter opens the exciting possibility that this method of using small nuclei might be used in nanoparticle or even molecular systems, where great advances are being made using femtosecond x-ray spectroscopy (*62*, *63*).

## MATERIALS AND METHODS

### Experiments

We used sterically stabilized PMMA spheres with a diameter of 2.0 μm and polydispersity of 4% as determined using static light scattering. The particles were dispersed in two different solvent mixtures. One consisted of cyclohexyl bromide (CHB) and cis-decalin and the other one of CHB, tetralin, and decalin. In both, 4 mmol of tetrabutyl ammonium bromide salt was dissolved to screen residual charges on the surface of the particles and avoid softness in the interaction potential. By comparing the radial distribution function to hard-sphere computer simulations, we found that our particles are very similar to hard spheres and that the choice of the solvent components has no measurable influence on the outcome of our analysis.

Nevertheless, we favor the three-component mixture because it enables us to match the density and refractive index of the particles independently. We exclusively investigated samples with careful density matching to avoid any influence of sedimentation on the nucleation events (*39*, *41*). The PMMA spheres were left in the solvent for a week to equilibrate, loaded to a glass capillary, and then imaged by 3D confocal laser scanning microscopy to obtain particle-resolved information.

### Particle tracking

The investigation of size distributions and structure of precritical nuclei places great demands on the quality of our experimental data. Missed particles and positions with a large localization error not only lead to a higher uncertainty in the effective volume fraction but also could shift the calculated nucleation barrier. Therefore, to extract particle coordinates from the experimental image, we use a recently developed deep-learning routine called Colloidoscope (*46*). In particular, in the regime of high volume fractions where particles are densely packed and their intensity distributions overlap, Colloidoscope tracks a higher number of particles with a smaller localization uncertainty in comparison to conventional approaches, based, for example, on the Crocker-Grier algorithm (*49*). This was determined using both simulated image data and detailed structural comparison between experimental data and simulated coordinates. We compared the outcome of different tracking routines by analyzing the number of tracked particles, the shape of the *g*(*r*) and the population of higher-order clusters. We found that Colloidoscope tracks more particles and produces a higher first peak in the *g*(*r*) and an increased higher-order cluster population, which indicates that the particles are tracked with better precision. In addition, Colloidoscope is particularly robust against the influence of photobleaching, allowing us longer exposure times and higher resolution for a longer period of time. From comparison to computer simulations, we found that Colloidoscope tracks ~98% of the particles in the experimental image with an error in position of around 5% of the particle diameter.

### Computer simulations

To compare our experimental results to ideal hard spheres, we perform both unbiased and biased simulations of size polydisperse hard-sphere mixtures. We match the experimentally observed distribution of particle diameters by using a deterministically generated Gaussian distribution with 4% polydispersity (defined as the ratio of the SD of the particle size σ over the mean particle size $\bar{\sigma }$). For the unbiased simulations, we use event-driven molecular dynamics simulations (*50*) of $N=10^{5}$ particles starting from a fluid phase for a range of different volume fractions ϕ in the supersaturated regime.

To facilitate comparison with the experimental data, we also recalculate the expected distributions of sizes of nuclei and TCC cluster concentrations in the presence of experimental errors. To mimic the experiments as closely as possible, we include the effects of a finite imaging volume, error in the position of the particles, and “missed” particles during tracking. First, we divide each simulation box of $N=10^{5}$ particles into subblocks of equal volume containing approximately $N_{\text{sub}}=5000$ particles to simulate the effects of a finite imaging volume on the detection of nuclei. Second, we apply a random displacement to all particle positions, drawn from a Gaussian distribution with zero mean and a SD of $d_{\text{err}}=0.05$. Third, we delete a small fraction of the particles from the configuration (typically less than 2%), with the particles chosen at random. Note that this approach does not take into account the possibility of correlations between tracking error and particle environments, which could, in principle, be present in the experimental data.

We also perform biased Monte Carlo simulations in the isobaric-isothermal ensemble with an umbrella sampling scheme (*52*), using the number of particles *n* in the largest crystalline nucleus as our bias parameter. The nucleus size was determined on the basis of standard bond-orientational order parameters, described in the following subsection. Note that the role of the bond-orientational order parameters on the height and shape of the free-energy curve was explored in (*23*). The umbrella sampling simulations allow us to reconstruct the free-energy barriers as a function of nucleus size at different supersaturations.

We also considered a hard-core Yukawa potential, taking into account softness from the screened residual charges on the surface of the particles. The pair potential is given by

$$\beta u\left(r\right)=\left\{ \begin{array}{cc} \text{βϵ}\frac{\text{exp}\left[-\text{κσ}\left(r/\sigma -1\right)\right]}{r/\sigma }, & r>\sigma \\ \infty , & r<\sigma \end{array}\right.$$ (4)

where βϵ is the value of the contact potential, κ denotes the inverse Debye screening length, and σ is the particle hard-core diameter. In this work, we chose βϵ = 11 and 1/κσ = 0.05 and mapped the state point to hard spheres using the Barker-Henderson effective hard-sphere diameter (*64*).

### Order parameter

We monitor the distribution of sizes of crystalline nuclei using the $q_{6}$ bond-orientational order parameters of ten Wolde *et al.* (*51*), for which we use the dot-product cutoff value to consider pairs of particles to be connected by a solid-like bond to be $q_{6}\left(i\right)\cdot q_{6}\left(j\right)\geq 0.7$ and where we consider a particle to be in a crystalline environment when its number of solid-like neighbors is at least 5. Specifically, we determine nearest neighbors using the SANN algorithm (*65*), which avoids the need for another cutoff parameter.

### Detecting higher-order structures

We analyze our data with the TCC (*47*). This detects geometric motifs whose bond topology is identical to that of clusters that minimize the local free energy for hard spheres in dense fluids (*54*). The TCC uses Voronoi tessellation to identify nearest neighbors and then detects shortest path rings (three, four, and five particles)in those configurations. Basic clusters are constructed on these, and, by adding more particles, clusters of various size can be identified. We use identical methods for analyzing experiments and computer simulations to prevent biasing certain cluster type populations.

### Asphericity calculation

We use PCA to quantify the sphericity of crystal nuclei. We apply PCA on sets of 3D coordinates in a crystal arrangement to find the two axes of our crystal nuclei with the highest ($\lambda_{\text{max}}$) and lowest variance ($\lambda_{\text{min}}$). Here, λ denotes the eigenvalues of the covariance matrix. For a perfect spherical nucleus, the two variances should have a similar value, and, therefore, $\lambda_{\text{min}}/\lambda_{\text{max}}$ should be close to one. For our data, we find distributions with a peak at 0.2 in computer simulations and experiments. This is likely because we only consider relatively small numbers of particles per precritical crystal nuclei. We also analyzed larger crystal nuclei of up to 100 particles that were produced with biased computer simulations and found that the ratio of variances slowly converges toward one with increasing nucleus size.
