# Supplementary material for: Narrowing down the cause of the hard-sphere nucleation discrepancy: The free energy of precritical nuclei is consistent with predictions
Source: Sci Adv. 2026 Apr 24;12(17):eaec8906. doi: 10.1126/sciadv.aec8906 (PMC13108528; doi:10.1126/sciadv.aec8906)
Supplement: Supplementary file 1 — Supplementary Text Figs. S1 and S2 [file sciadv.aec8906_sm.pdf]

Science Advances
AAAS

Supplementary Materials for

Narrowing down the cause of the hard-sphere nucleation discrepancy: The free energy of precritical nuclei is consistent with predictions

Lars Kürten *et al.*

Corresponding author: C. Patrick Royall, paddy.royall@espci.psl.eu

Sci. Adv. **12**, eaec8906 (2026)
DOI: 10.1126/sciadv.aec8906

This PDF file includes:

Supplementary Text
Figs. S1 and S2

Mapping the State Point Between Experiment and Simulation

Nucleation barriers are highly sensitive to the state point of the system. We want to calculate the start of the barrier from the size distribution of precritical nuclei found in experiments and compare to computer simulations. Therefore, special attention must be paid to the determination of effective volume fractions in experiments. In the following a newly developed procedure of carefully mapping experimental data sets onto computer simulations is described. One advantage of this technique is that almost no *a priori* knowledge of the properties of the sample is required for the complete characterisation. An experimental data set of a hard spheres system is described by a few parameters. These include the polydispersity, the effective volume fraction, the localization uncertainty, and the fraction of particles that get lost during the tracking process.

The polydispersity was determined from static light scattering experiments. The localisation uncertainty of the tracking routine can be inferred from its effect on the shape of the first peak of the radial distribution function and was determined in a previous publication [46]. We found that adding a Gaussian error with 5% standard deviation to the simulation coordinates matches the experimental conditions.

For the mapping we use two observables, both sensitive to the state point of the system and the interaction potential of the particles. The first one is the logarithm of the total correlation function $\log(|rh(r)|)$ with $h(r) = g(r) - 1$ (Fig. S1). The decay of the height of the peaks is determined by the state point of the system. The second quantity is the population of higher order clusters, identified by the TCC (topological cluster classification) [47]. Both observables are also influenced by the properties of the tracking routine in a way similar to that of a change in the state point. That makes the mapping somewhat subtle.

In general, we want to find the effective volume fraction of the experiments, the effective average hard sphere diameter of the particles and from that an estimate of the fraction of particles not being identified by the tracking algorithm. The mapping routine consists of 3 steps. The first step is a comparison of the total correlation function. The experimental data is binned onto the available simulation state points. The result is a first rough estimate of the effective volume fraction for the experiments.

The second step is a careful comparison of populations of specific higher-order clusters that have no similarities to crystal nuclei. In comparison to the pair correlation function, cluster populations are more sensitive to the state point and allow a more accurate second estimation of the effective volume fraction.

The last step deals with the fact that for experimental data we are only considering a subset of coordinates of the "true" system. We again compare the total correlation functions of experiments and computer simulations based on the results from the second step. From that comparison an effective hard sphere diameter σ_{eff} can be extracted that is used to calculate a second effective volume fraction from the number of particles tracked N_p.

$$\phi_{\text{exp}}^{\text{counted}} = N_p \frac{\pi\sigma^3}{6V} \tag{S1}$$

Here V is the imaged volume of the experimental system. This second estimate is smaller compared to the true value ($\phi_{\text{exp}}^{\text{counted}} < \phi_{\text{exp}}^{\text{true}}$) due to particles lost during the tracking routine. To ensure that we actually compare

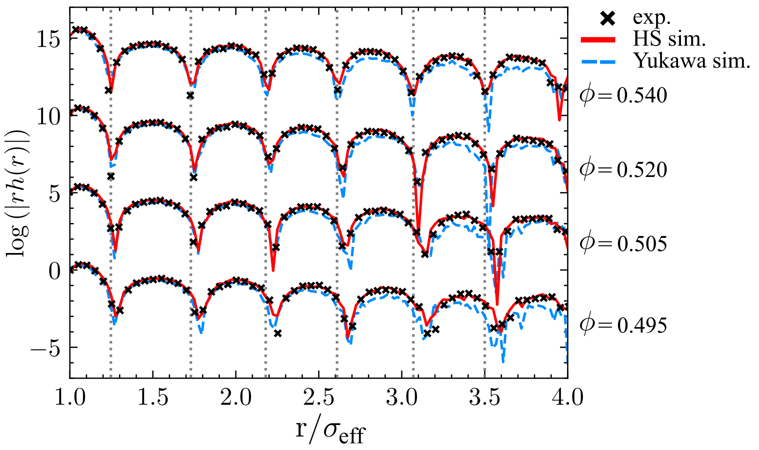

FIG. S1. **Total Correlation Function**. Total correlation function at a range of volume fractions in coexistence regime. Data are offset for clarity.

the identical effective state points we delete the same fraction of particles from the simulation coordinates, so that $\phi_{\text{exp}}^{\text{counted}} = \phi_{\text{sim}}^{\text{counted}}$. The fraction of deleted particles varies slightly for different volume fractions around 1%. A comparison of the distribution of those effective volume fractions is shown in the insets of Fig. S2.

Barriers

The outcome of the bond-orientational order parameter analysis is a discrete value of the free energy barrier for a certain nucleus size. These discrete values were binned manually to ensure a reasonable representation of the raw data (Fig. S2). The size of the bins varies according to the number of nuclei found for each size. The error bar is then calculated as the square root of the number of nuclei in each bin.

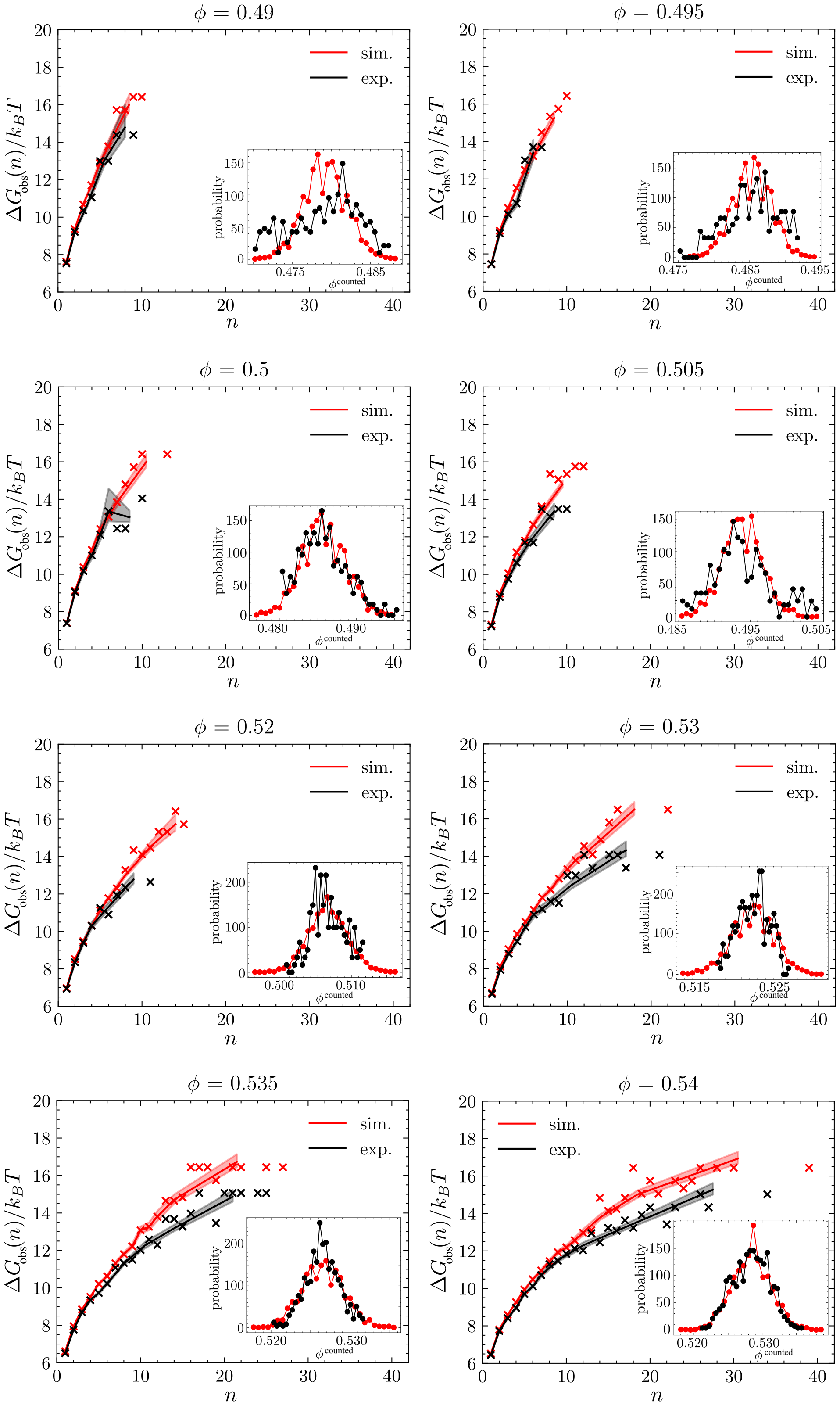

FIG. S2. **Effective Nucleation Barriers**. Raw output of the bond-orientational order parameter analysis (x-symbols) and the effective and binned nucleation barrier. Insets show a comparison of the effective volume fraction by counting particles between experiments and simulations.
